# Ameliorative Effects of Silicon against Salt Stress in *Gossypium hirsutum* L.

**DOI:** 10.3390/antiox11081520

**Published:** 2022-08-04

**Authors:** Leilei Li, Qian Qi, Hengheng Zhang, Qiang Dong, Asif Iqbal, Huiping Gui, Mirezhatijiang Kayoumu, Meizhen Song, Xiling Zhang, Xiangru Wang

**Affiliations:** 1 State Key Laboratory of Cotton Biology, Institute of Cotton Research of Chinese Academy of Agricultural Sciences, Anyang 455000, China; 2 Western Agricultural Research Center of Chinese Academy of Agricultural Sciences, Changji 831100, China

**Keywords:** salinity, silicon, chloroplast ultrastructure, antioxidant enzymes, photosynthetic performance

## Abstract

Silicon (Si) could alleviate the adverse effect of salinity in many crops, but the effect in cotton remains unclear. In this study, we evaluated the role of Si in regulating the salt stress tolerance of cotton by analyzing the induced morpho-physiological changes. A hydroponic experiment was conducted by using contrasting salt-tolerant cotton genotypes (sensitive Z0102; tolerant Z9807) and four treatments (CK, control; CKSi, 0.4 mM Si; NaCl, 150 mM NaCl; NaClSi, 150 mM NaCl+0.4 mM Si). The results showed that Si significantly enhanced the net photosynthesis rate and improved the growth of cotton seedling under salt stress in both salt-sensitive and salt-tolerant genotypes. Exogenous Si significantly reduced the accumulation of reactive oxygen species (ROS) and decreased the malondialdehyde (MDA) content in salt-stressed cotton. In addition, the application of Si up-regulated the expression of *CAT1*, *SODCC* and *POD*, and significantly enhanced the antioxidant enzymatic activities, such as catalase (CAT) and peroxidase (POD), of the salt-stressed cotton seedlings. Further, Si addition protected the integrity of the chloroplast ultrastructure, including key enzymes in photosynthesis such as ferredoxin-NADP reeducates (FNR), ATP synthase (Mg^2+^Ca^2+^-ATPase) and ribulose-1, 5-bisphosphate carboxylase/oxygenase (RubisCO), and the structure and function of the photosynthetic apparatus PSII from salt stress. Moreover, Si significantly increased the effective stomatal density and stomatal aperture in the salt-stressed cotton seedlings. Taken together, Si could likely ameliorate adverse effects of salt stress on cotton by improving the ROS scavenging ability and photosynthetic capacity.

## 1. Introduction

Crop growth and yield formation are affected by various biotic and abiotic stresses. Soil salinity is one of the major constraints contributing to a primary limit on crop productivity. Salt stress could cause osmotic imbalance and ion toxicity in plants, following by the inhibition of mineral nutrition absorption and transport [1,2]. It also degrades the photosynthetic pigments such as chlorophyll and carotenoids, limited Ribulose-1, 5-bisphosphate carboxylase/oxygenase activity, destructs the photosynthetic apparatus [3,4,5], and consequently leads to poor photosynthesis and a decline in yield [6,7]. Salt stress could cause varied degrees of yield reduction for different crops, and the yield reduction increases with the intensification of the stress level [8]. Unfortunately, more than 20% of the world’s cultivated land is salinized and this proportion may increase to 50% by 2050 due to climate change and irrational water use for agricultural irrigation [9]. Therefore, strategies to alleviate the deleterious effect of salinity are urgently needed.

Silicon (Si) is the second most abundant mineral element next to oxygen on the earth’s surface and was included as a “quasi-essential” element by the International Plant Nutrition Institute recently [10]. Si has a positive role in the growth and development of many crops, especially the gramineous species including wheat, rice, maize, and barley, which are considered as Si accumulators [11,12,13]. Many recent studies have proved that Si has a beneficial effect on improving plant resistance to disease and insect pests [14]. Si can also help to mitigate the deleterious impact of abiotic stress, such as heavy metals [15,16,17], herbicides [18], drought [19], and also salinity [20]. Since plant roots only absorb mono silicic acid (Si (OH)_4_, pH < 9), and Si in the soil is mainly in the form of silicate or aluminum silicate, exogenous Si as fertilizer is applied despite the abundant Si in soil [11,21]. To date, the positive effect of Si on plant growth under stress is confirmed, but the exact mechanisms are still under debate and need further studies [22].

Reactive oxygen species (ROS) is a multifunctional signal that can be induced by various environmental pressures including salinity. Low concentration of ROS can be used as a signal molecule to regulate plant growth and development, but excessive accumulation has a negative impact on plants, which gives rise to the destruction of cell structure and accelerates cell death in plants [23]. Therefore, plants usually maintain the homeostasis of intracellular ROS through antioxidant defense systems (including antioxidant enzymes like superoxide dismutase (SOD), peroxidase (POD), catalases (CAT), etc., and non-enzymatic components as ascorbate and glutathione). Several studies reported that Si supplementation under salt stress plays a positive role in reducing ROS levels and enhancing the stability of lipids in cell membranes, but the antioxidant mechanisms differ between plant species. It has been reported that the application of Si to salt-stressed barley significantly increased SOD activity in leaves, and SOD and CAT activity in roots [24]. The results of Zhu et al. [25] showed that adding Si to a salt-treated cucumber caused an increase in SOD activity in the leaves, but an increase in CAT was not observed. Moreover, the effects of Si on antioxidant enzymes activity were related to variety and stress duration [25].

Photosynthesis is the primary metabolic process of plants that provides materials and energy for growth and development. Studies found that Si could enhance the photosynthesis of plants growing under stress including salinity [26]. The deposition of Si in leaves and stems improves the mechanical strength and uprightness of plant tissues, so that plants have better light-receiving posture and light-receiving area, thereby improving photosynthesis [27]. In addition to the mechanical effects of Si, the exogenous application of Si could increase the photosynthetic pigment as chlorophyll in wheat, rice, and tomato under salinity conditions, and ensure the normal photosynthetic rate of leaves [18,20,28]. Kang et al. [29] and Sattar et al. [30] found that Si significantly increased the stomatal conductance and thus enhanced the photosynthetic rate in saline conditions. Furthermore, studies on tomato plants indicated that Si mitigates the adverse effect of salt stress on photosynthesis by protecting the photosynthetic apparatus and increasing the PSII efficiency [31]. Given that the different effects of Si on photosynthesis-related physiological processes vary across different plants, more systematic and comprehensive studies are still needed.

Cotton (*Gossypium hirsutum* L.) is one of the most important cash crops and provides raw materials for the textile industry. Due to higher tolerance to salinity, cotton is recognized as a pioneer crop in the development of saline-alkali land [32]. However, high salt concentration inhibits cotton growth and development, and can finally cause severe yield reduction. At the present time, a substantial amount of studies considering the alternative effect of Si on eliminating salt stress have been conducted in crops like wheat, rice, and maize [18,28]; however, studies regarding the application of Si to improve salt tolerance in cotton are still to be elucidated. Therefore, in this study, we conducted a hydroponic experiment to find out the alleviative effect of Si on cotton growth, antioxidant enzymatic activities, photosynthetic parameters, chlorophyll content, chlorophyll fluorescence parameters, and chloroplast ultrastructure under salinity stress. We hypothesize that Si alleviates oxidative damage induced by salt stress by enhancing photosynthetic capacity and antioxidant enzyme activity, thereby promoting cotton growth. The findings can provide theoretical support for the development of cotton planting technology in salt-alkali lands.

## 2. Materials and Methods

### 2.1. Plant Materials and Growth Conditions

In the current experiment, two cotton genotypes, one being salt-tolerant (Z9807) and the other salt-sensitive (Z0102), [7] were grown in the hydroponic culture at the Cotton Research Institute of the Chinese Academy of Agricultural Sciences, Anyang, China. Healthy and uniform sized seeds were surface sterilized for 5 min with ethyl alcohol (75% *v*/*v*), then disinfected with 2.5% (*v*/*v*) sodium hypochlorite solution for 15 min, followed by rinsing with double-distilled water five times. Afterward, the seeds were sown into a germination pot containing sterilized vermiculite. After seven days of germination, uniform and healthy seedlings were transplanted to 10 L plastic boxes, wrapped in foam strips at the root-shoot junction, and fixed in holes of a black sheet, containing half-strength modified Hoagland nutrient solutions [7]. The seedlings were grown in the greenhouse under 14/10 h light/dark cycle, 28 ± 2 °C /22 ± 2 °C day/night temperatures, 60% humidity, respectively, for the entire growth period. The nutrient solution was changed every week to avoid nutrient depletion and secondary metabolic damage. When the cotton seedlings grew to three true leaves (about two weeks after germination), the following four treatments were applied for each cultivar: CK (normal conditions, 1/2-strength Hoagland nutrient solution), CKSi (CK+0.4 mM Si), NaCl (CK+150 mM NaCl), and NaClSi (CK+150 mM NaCl+0.4 mM Si). The amount of Si was based on the previous screening of Si concentration (data not shown). Before NaCl treatment, the plants of CKSi and NaClSi treatments were pretreated with 0.4 mM Si (Na_2_O_3_Si·9H_2_O) for 7 days. To minimize salt shock, NaCl concentration was raised stepwise in aliquots of 50 mM every 6 h until the final salinity levels were achieved. The experiment included eight treatments with three replicates (12 plants per box). Moreover, to avoid the impact of micro-environmental effects, the plastic boxes were frequently moved to a new position. The consumed solution was re-added every two days to recover the target volume, and the pH of the nutrient solution was adjusted to 6.8 using diluted H_2_SO_4_ or KOH. The culture mediums were renewed every 7 days. After 14 days of treatments, cotton seedlings were harvested, and the relevant parameters were analyzed. The fourth fully-expanded leaf from the top of the cotton plant was used for physiological traits determination, RNA extraction, and photosynthetic structure observation.

### 2.2. Measurement Traits and Methods

#### 2.2.1. Plant Growth and Biomass Accumulation

After 14 days of treatment, the plant height from the cotyledon to the apex was measured. The seedlings were separately harvested and divided into shoot and root and placed in the oven for 0.5 h at 105 °C, followed by 80 °C for the next 48 h until constant weight. Thereafter, the dry weight of the root and shoot was recorded.

#### 2.2.2. Measurement of Gas Exchange Parameters

Leaf gas exchanges of different treated cotton plants were determined with a LI-6800 portable photosynthetic system (LICOR, Lincoln, NE, USA) at 8:30–11:30 am. During the measurements, the photosynthetic photon flux density was 200 µmol s^−1^, the CO_2_ concentration was 400 µmol mol^−1^, and the relative humidity was 55%. The gas exchange parameters such as net photosynthetic rate (Pn), stomatal conductance (Gs), transpiration rate (Tr), and intercellular CO_2_ concentration (Ci) were recorded from six seedlings per treatment simultaneously.

#### 2.2.3. Determination of ROS and Antioxidant Enzymes

The histochemical detection of H_2_O_2_ and O_2_^−^ in cotton leaves was conducted by using DAB and NBT methods, respectively, and the content was detected by using the corresponding kits according to the manufacturer’s instructions (Solarbio, Beijing, China). Malondialdehyde (MDA) content was determined by the thiobarbituric acid (TCA) reactions.

To measure the antioxidant enzymes activity, approximately 0.5 g frozen leaf sample was weighed and ground in liquid nitrogen, meanwhile 10 mL 50 mmol·L^−1^ sodium phosphate buffer (pH 7.8) was added containing 1% polyvinyl pyrrolidine, 0.2 mmol·L^−1^ ethylenediamine tetraacetic acid, and 10 mmol·L^−1^ magnesium chloride, and was then centrifuged at 12,000× *g* for 20 min at 4 °C. The supernatant was collected, stored at 4 °C, and used to determine the activity of POD, SOD, and CAT following Jin et al. [33].

#### 2.2.4. Measurement of Photosynthetic Pigments

Approximately 0.1 g of fresh leaf tissue was extracted with 95% ethanol, and was then placed in the dark place for 48 h (shaking every 12 h) until the leaves turned white. The absorbance of the extract solution of all samples was analyzed using a spectrophotometer (UV-1280, Shimadzu, and Kyoto, Japan) at wave lengths of 660 nm, 649 nm, 470 nm, and calculated according to Sikder et al. [7].

#### 2.2.5. Assay of Chlorophyll Fluorescence Parameters

Chlorophyll fluorescence was determined via Plant Efficiency Analyzer (Handy PEA, Hansatech, UK). After 30 min of dark adaptation, the leaves received continuous illumination of 3000 μmol m^−2^ s^−1^ of red light to induce a fast chlorophyll fluorescence curve. Instantaneous fluorescence from 10 μs to 300 s was recorded, and then an OJIP curves diagram was plotted with the logarithm of the measurement time. Each treatment had three biological replicates with nine plants per replicate.

#### 2.2.6. Activity of Ferredoxin-NADP Reductase (FNR), ATP Synthase and Ribulose-1, 5-Bisphosphate Carboxylase/Oxygenase (RubisCO)

A total of 0.1 g of fresh leaf power was homogenized with 1 mL of 50 mM Tris–HCl buffer solution (pH 8.0). The homogenate was centrifuged at 8000× *g* for 10 min at 4 °C. Mg^2+^Ca^2+^-ATPase was determined with the Mg^2+^Ca^2+^-ATPase activity detection kit (Art. NO. BC0965, Solarbio, Bejing, China) according to the manufacturer’s instructions.

The activities of RubisCO and FNR were determined by ELISA. We took 0.1 g of fresh leaves and added 0.9 mLof PBS (pH 7.5, containing 137 mM NaCl, 1.4 mM KH_2_PO_4_, 8.3 mM Na_2_HPO_4_, and 6.3 mM Tris-HCl) for ice bath homogenization. The homogenate was centrifuged at 4000 rpm for 20 min at 4 °C. Rubisco ELISA kit (Art. NO. ml022780, MLBio, China) and FNR ELISA kit (Art. NO. MM-6279402, Mmbio, China) were used to assess the corresponding enzyme.

#### 2.2.7. Estimation of Stomatal State

Leaf samples were dehydrated in a graded series of ethanol (30, 50, 70, 90, 95, and 100%-anhydrous), following the addition of pure isoamyl acetate, and the critical point was dried by flooding with liquid CO for 2 h, and then we increased the temperature and pressure to critical point. The samples were sputter-coated with gold and examined with a scanning electron microscope (SU3500, Japan) to observe the stoma state in a specific field of view (1000× magnification for stomata density; 3000× magnification for single stomata traits). Effective stomata density (number of opening stomata mm^−2^) was calculated based on nine views. Furthermore, 15 opening stomata of each treatment were used to determine the stomatal pore length, width, and surface (to indicate the stomata opening) by using ImageJ software (National Institutes of Health, Bethesda, MD, USA).

#### 2.2.8. Transmission Electron Microscope Assay for Chloroplast Ultrastructure

The chloroplast ultrastructure was studied according to Shu et al. [34] with small modifications. Fresh leaves were cut into 2 × 2 mm and placed immediately in 2.5% (*w*/*v*) glutaraldehyde, and then they were degassed and fixed under a vacuum for 24 h at 4 °C. After washing with 0.1 mM sodium phosphate buffer (pH 7.4), they were post fixed in 2% (*w*/*v*) osmium tetroxide for 4 h. Then the samples were rinsed three times with sodium phosphate buffer for 15 min. Rapid dehydration was achieved by placing the samples in 30% ethanol for 15min, followed by changes in 50%, 70%, 90%, and 100% ethanol (15 min each). The specimens were then placed in epoxy resin to infiltrate, embed, and polymerize. After that, they were stained with uranyl acetate and lead citrate sequentially before being examined under a transmission electron microscope (Hitachi Model H-7800 TEM, Carl Zeiss, Göttingen, Germany).

#### 2.2.9. Quantitative Real-Time PCR Analysis

DP441 plant total RNA extraction kit from TIANGEN Company (Beijing, China) was used to extract total RNA from cotton seedling leaves, and 1 μL of RNA sample was taken to detect the concentration of extracted RNA, and then the RNA was diluted to the same concentration with RNase-Free ddH_2_O. For all samples, 1 μL of RNA was used for reverse transcription synthesis of cDNA according to the protocol (TaKaRa RR036A).

The expression of genes involved in the ROS scavenging and RubsiCO was determined by conducting quantitative real-time PCR (qRT-PCR). The relative gene expression was determined by the comparative CT method, also referred to as the 2^−ΔΔCT^ methods, and three biological replicates were performed for each gene. Specific primers were based on the gene sequences of the NCBI GenBank database (https://www.ncbi.nlm.nih.gov (accessed on 15 September 2021)) and the Cotton Functional Genomics Database website (https://cottonfgd.org/ (accessed on 31 December 2021)). The Actin gene was used as the internal reference and the primer sequences were shown in Table S1.

### 2.3. Statistical Analysis

Experimental data were analyzed using SAS software (SAS 9.4, Cary, NC, USA), and significant differences among treatments were compared using the least significant difference (LSD) test at the 5% levels of probability. Origin 2021b (2021, OriginLab Corporation, Northampton, MA, USA) was used for plotting graphs.

## 3. Results

### 3.1. Effect of Exogenous Si on the Growth of Cotton Seedlings under Salt Stress Subsection

**Figure 1:**
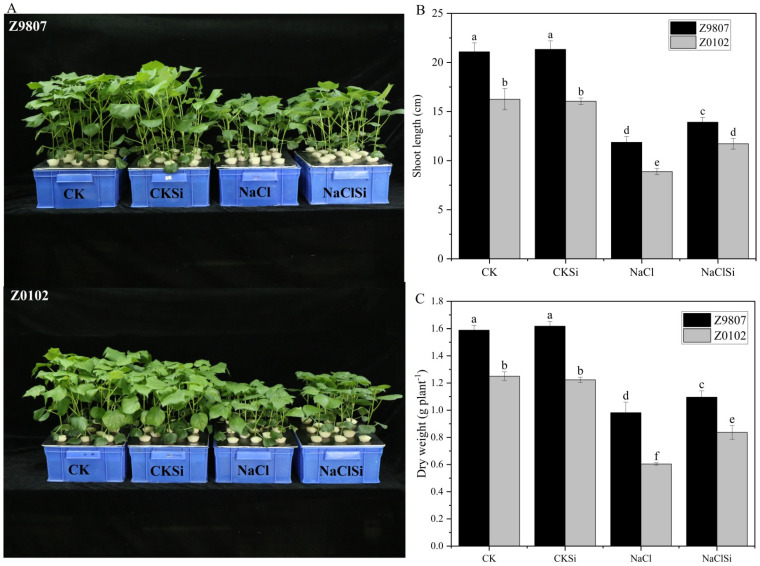
Effects of Si on cotton growth (**A**), shoot length (**B**), and biomass accumulation (**C**) at 14 days after various treatments. Error bars represent the standard error (*n* = 3). Different letters mean significantly different among treatments at *p* ≤ 0.05.

The growth of cotton seedlings was obviously different among the treatments (Figure 1). Salt stress significantly inhibited the cotton growth and caused notable decreases in shoot length and biomass accumulation. Salt stress significantly reduced the shoot length and dry weight of Z9807 by 43.6% and 38.2%, and the reduction was higher in salt-sensitive genotype Z0102 as 46.0% and 51.7%, respectively. Compared with only the salt-treated plants, all of these growth parameters of Si-treated plants were improved in both cotton genotypes, indicating a positive effect of Si on enhancing salt tolerance of cotton.

### 3.2. Regulation of Gas Exchange Parameters upon Exogenous Silicon

Salt stress caused a significant reduction in photosynthetic rate (Pn), intercellular CO_2_ concentration (Ci), transpiration rate (Tr), and stomatal conductance (Gs) in both genotypes; however, the adverse effect of salinity was alleviated with the application of Si (Table 1). The addition of Si had no significant effect on the Pn, Ci, Tr, and Gs of Z9807 under non-stress conditions. Unlikely, exogenous Si significantly improved Tr, Ci, and Gs in Z0102.

### 3.3. Effect of Si on the Accumulations of H_2_O_2_, O_2_^−^ and MDA in Cotton Seedlings under Salt Stress

**Figure 2:**
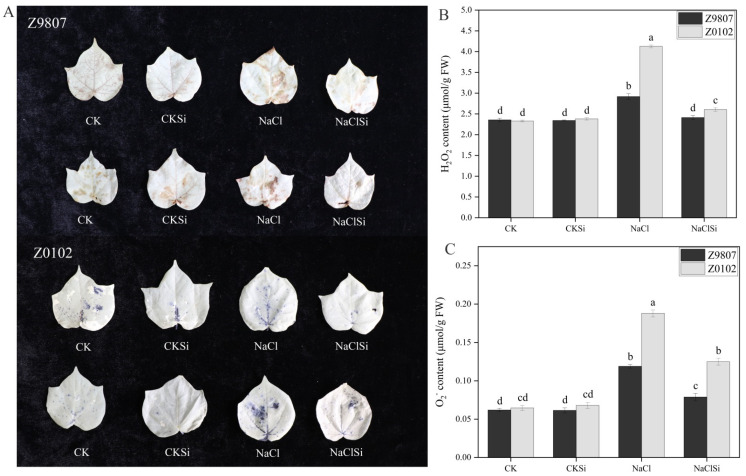
(**A**–**C**) Detection of hydrogen peroxide (H_2_O_2_) and superoxide anion (O_2_^−^) accumulation in the leaves of Z9807 and Z0102 under different treatments. DAB can be oxidized by H_2_O_2_ to form a reddish-brown precipitate. NBT reacted with O_2_^−^ to produce dark blue insoluble compounds. Error bars represent the standard error (*n* = 3). Different letters mean significantly different among treatments at *p* ≤ 0.05.

In order to find out whether Si-promoted cotton growth under the salt condition was related to the alleviation of salt-induced oxidative stress, we carried out NBT and DBS staining and further determined the content. As shown in Figure 2, salt stress led to a significant increase in ROS accumulation in cotton leaves. The content of H_2_O_2_ and O_2_^−^ in Z9807 seedlings grown under salt stress significantly increased by 23.8% and 92.3% compared with the control cultivar, and the increase in Z0102 was much higher as 177.3% and 190.6%, respectively (Figure 2B,C). The application of Si did not affect the H_2_O_2_ or O_2_^−^ level under the non-salinity condition, but significantly decreased it in salt-stressed plants (Figure 2B,C).

**Figure 3:**
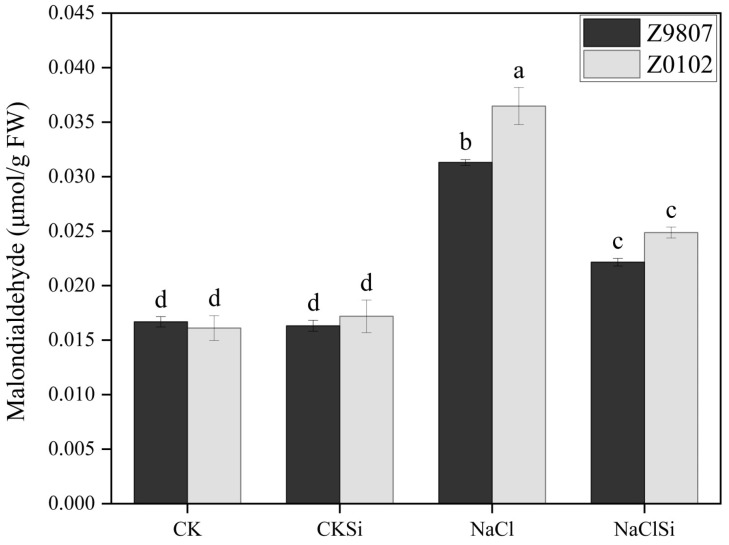
Effect of Si on MDA content in cotton leaves under salt stress. Error bars represent the standard error (*n* = 3). Different letters mean significantly different among treatments at *p* ≤ 0.05.

MDA is the final decomposition product of membrane lipid peroxidation, and its content can reflect the degree of plant injury. Our results showed that salt stress induced the accumulation of MDA in cotton leaves (Figure 3). The salt-induced MDA increase in the salt-sensitive genotype (126.7%) was higher than the salt-tolerant genotype (87.4%). Si supplementation significantly decreased the MDA content in Z9807 and Z0102 by 29.4% and 32.1% in comparison with the salt treatment (Figure 3).

### 3.4. Effects of Exogenous Si on Antioxidase Activity and Antioxidase Gene Expression

**Figure 4:**
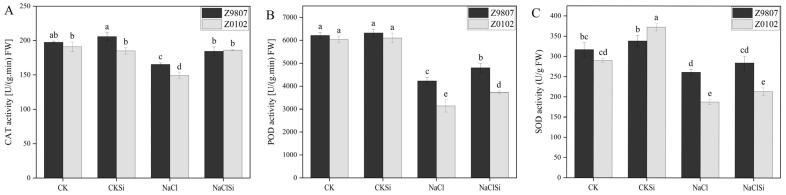
Effect of Si on the activity of CAT (**A**), POD (**B**), and SOD (**C**) under saline and non-saline conditions. Error bars represent the standard error (*n* = 3). Different letters mean significantly different among treatments at *p* ≤ 0.05.

Salt stress induced a significant decline of CAT, POD, and SOD activity in both genotypes. However, the adverse effect of salinity on antioxidant activity was significantly relieved by exogenous Si. Upon Si supplementation in salt-stressed plants, the CAT, POD, and SOD activity was 30.4%, 18.8%, and 11.6% higher in Z0102, whereas it was 11.2%, 13.5%, and 1.6% higher in Z9807 compared with the salt-treated seedlings alone (Figure 4). Compared with the control, Si application had no significant effect on the antioxidant enzymatic activity except for the improved SOD of Z0102 (Figure 4).

**Figure 5:**
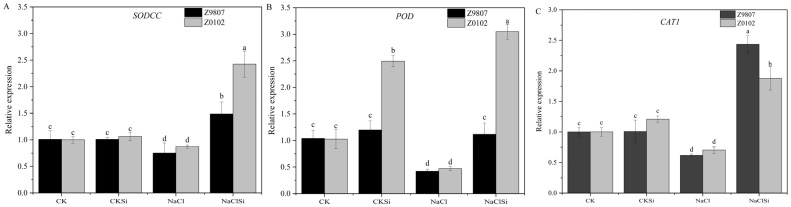
Expression of *SODCC* (**A**), *POD* (**B**), and *CAT1* (**C**) in cotton functional leaf. Error bars represent the standard error (*n* = 3). Different letters mean significantly different among treatments at *p* ≤ 0.05.

To further explain the effect of Si on antioxidant capacity, we investigated the benefits of Si at the transcriptional level (Figure 5). On the whole, the relative expression levels of *SODCC*, *POD*, and *CAT1* were significantly down-regulated by salt at the 14 days of salt treatment. The application of Si significantly up-regulated the expression of antioxidant enzyme genes in leaves of salt-stressed cotton (except the POD of Z9807), especially in the salt-sensitive genotype Z0102. These results indicated that the exogenous Si increases the activity of POD and CAT by inducing gene expression of *POD* and *CAT1* under salinity.

### 3.5. Effects of Exogenous Silicon on Chlorophyll Content

Salt stress significantly reduced the chlorophyll a (Chl a), chlorophyll b (Chl b), chlorophyll a + b (TChl), and carotenoid contents of cotton seedlings, but the reductions were alleviated by Si application (Table 2). Compared with the salt treatment, the Si-pretreatment increased the TChl and carotenoids content of salt-stressed seedlings by 35.4% and 4.8% in Z9807, whereas 30.9% and 20.4% in Z0102, respectively (Table 2).

### 3.6. Exogenous Silicon Facilitates Stomatal Opening

**Figure 6:**
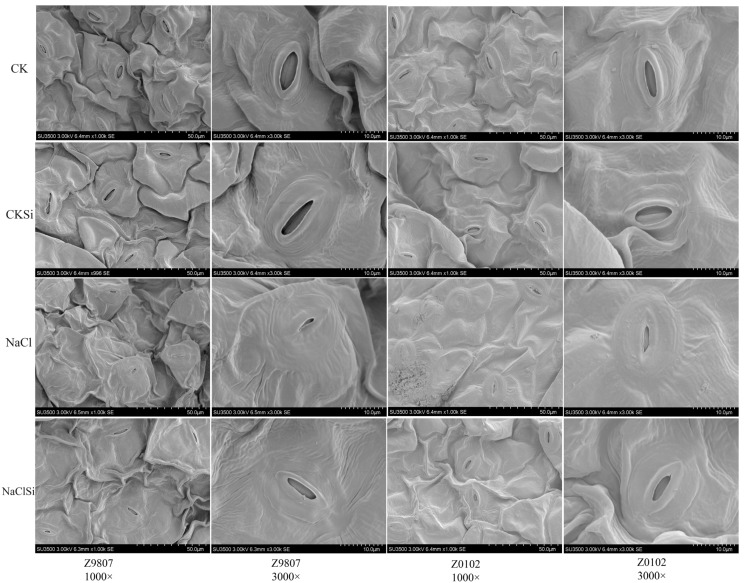
Effect of exogenous Si on the stomatal state of Z0102 and Z9807 leaves under saline and non-saline conditions. Comparison of the stomatal state was observed using a scanning electron microscope (SEM) image.

Stomata are the main channels for the exchange of water and air between plants and the external environment, and stomatal character is one of the important indicators reflecting the adaptability of plants to the ecological environment. The scanning electron microscopy showed that, under control conditions, the leaf stoma was normally developed and in an open state. However, under salt conditions, most of the stomata closed, and the effective stomata density significantly decreased by 53.0% to 63.2% in two genotypes (Figure 6, Table S2). In addition, the stomatal pore length and aperture of the opening stomata were decreased by 8.0% to 8.8% and 72.3% to 80.1%, respectively, by salt (Table S2). Under salt stress, Si application significantly promoted the opening of the stomata (Figure 6). The stomatal pore length, aperture, and ESD increased by 14.3% to 16.3, 134.4% to 205.7%, and 57.5% to 87.5%, respectively, after having been treated with exogenous Si compared with the salt treatment. In addition, under non-stress conditions, the addition of Si can also significantly improve the stomatal length and opening (Table S2).

### 3.7. Ultrastructure of the Chloroplasts

**Figure 7:**
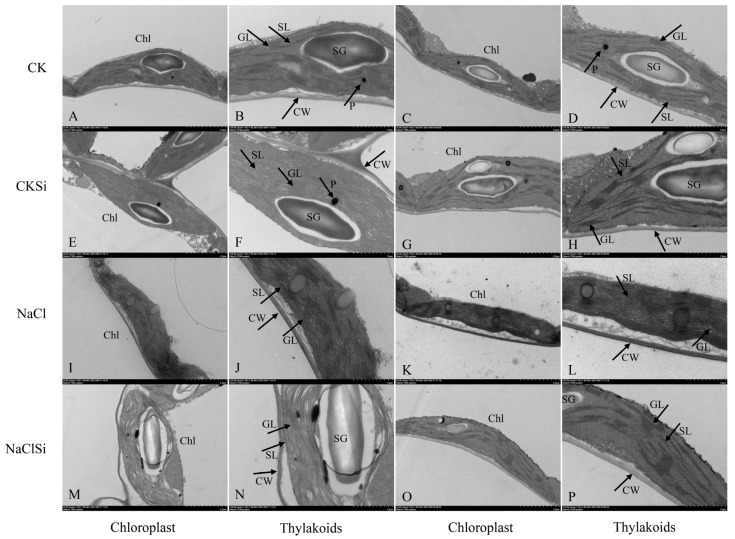
(**A**–**P**) Effect of exogenous Si on the ultrastructure of chloroplasts and thylakoid membranes of Z9807 and Z0102 leaves under saline and non-saline conditions. Scale bars: 2.0 μm (chloroplast); 1.0 μm (Thylakoids); Chl, Chloroplast; CW, cell wall; GL, Grana lamellae; SL, Stroma lamellae; SG, Starch grain; P, Plastoglobule.

Chloroplasts of non-salt-treated plants regardless of Si were well-developed, inseparable from the membrane, and contained a regular arrangement of stroma lamellae and extremely compact grana lamellae inside (Figure 7A–H). Salt stress caused pronounced changes in the structure of the chloroplasts and thylakoids when compared with the control. The chloroplasts of cotton plants under salt stress were separated from the cell membrane, particularly in the salt-sensitive genotype Z0102, and starch granules were significantly reduced or even disappeared in the salt-treated plants (Figure 7I–L). Moreover, salt stress induced severe disintegration of thylakoid membranes and led to the invisible stromal and granal thylakoids (Figure 7J,L). However, exogenous Si alleviated the damage of salt stress on chloroplasts. Si decreased the separation intensity of chloroplasts envelopes to the cell membrane and maintained a relatively well-preserved internal lamellar system in the chloroplasts of salt-stressed leaves (Figure 7M–P). Overall, exogenous Si could protect the chloroplast from the damage of salt stress to some extent.

### 3.8. Impact of Exogenous Si on the Fluorescence Rise Kinetics O-J-I-P Curves in Cotton Leaves under Salt Stress

**Figure 8:**
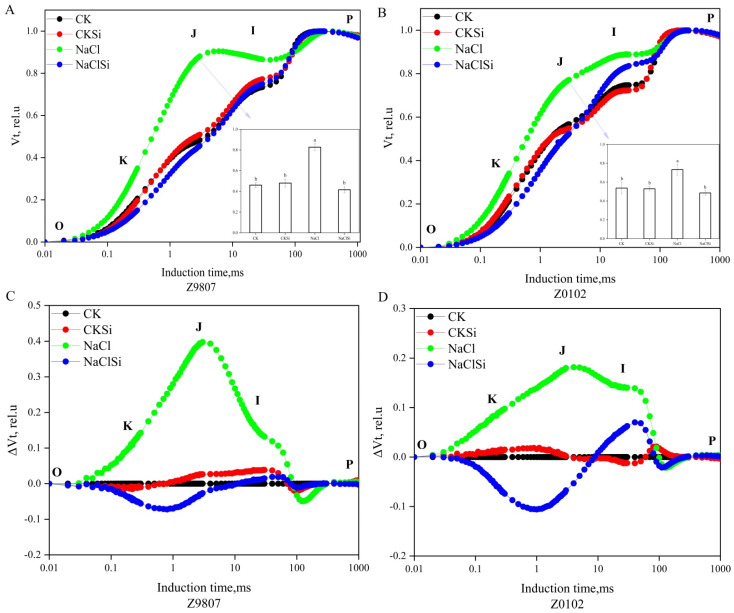
Effects of exogenous Si on relative chlorophyll a fluorescence [(**A**) (Z9807), (**B**) (Z0102), *V_t_* = (Ft − F0)/(Fm − F0)], and the relative differences of chlorophyll a fluorescence [(**C**) (Z9807), (**D**) (Z0102), Δ*V_t_* = *V_t_* (different treatments without CK) − *V_t_* (CK)] in third fully expanded leaves. Each curve is the average of three replicates. Different letters mean significantly different among treatments at *p* ≤ 0.05.

To decipher the effect of Si-mediated alleviation of salt stress on cotton, the impact of Si on the photochemistry of the photosystem II (PSII) was determined through a chlorophyll fluorescence transient-JIP test. The transient chlorophyll fluorescence rise kinetics of the third leaf from the top showed a typical OJIP transient on a logarithmic time scale. However, the shape of the OJIP fluorescence transient measured on cotton-only and treated with NaCl differed markedly from other treatments (Figure 8A,B). It was glaringly obvious to see that the salinity had a significant influence on the fluorescent transient, especially the J step (2 ms), which was significantly higher than the other treatments in both genotypes.

To further analyze the changes in the kinetics of the increase in the fluorescence of OJIP, we subtracted the doubly normalized fluorescence value of the control from the doubly normalized fluorescence value of other treatments, calculated it and then constructed the difference in the variable fluorescence curve (Δ*V_t_*) (Figure 8C,D). The results showed that the curves of the two genotypes were obviously deformed, and the K point (0.3 ms), J phase (2 ms), and I phase (30 ms) increased significantly compared to the control, whereas the exogenous Si slowed down the upward trend. These results indicated that the photosynthetic electron transport chain was disturbed under salt stress, which was able to be regulated with the exogenous Si pretreatment.

### 3.9. Fluorescence Transient OJIP-Detection Analysis

**Figure 9:**
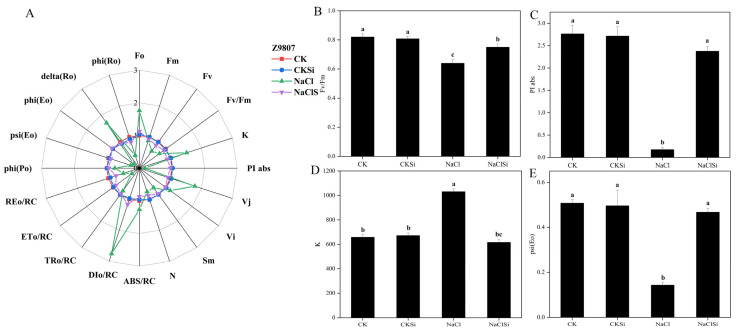
Analysis of selected JIP parameters derived from chlorophyll-a fluorescence in cotton genotype Z9807 under different treatments. (**A**) spider plot of a few fluorescence parameters of cotton leaves, and for each parameter, the value of control was set 1. (**B**) Fv/Fm. (**C**) fluorescence intensity of K point. (**D**) PIabs, performance index for energy conservation from photons absorbed by PSII until the reduction of intersystem electron acceptors. (**E**) Psi (Eo), the ratio of QA driven by exciton occupancy in excitons captured by the reaction center to reduce excitons to drive electron transfer to other electron acceptors in the electron transport chain beyond QA. Different letters mean significantly different among treatments at *p* ≤ 0.05.

**Figure 10:**
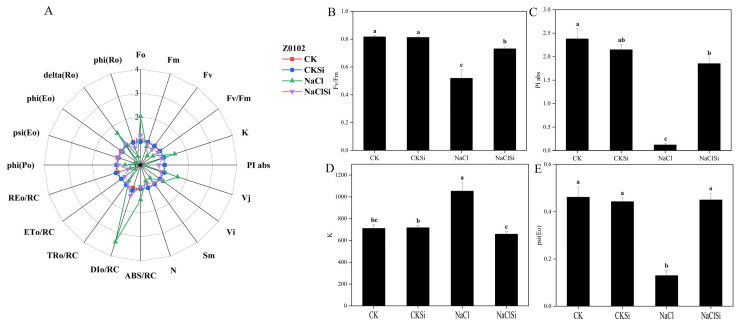
Analysis of selected JIP parameters derived from chlorophyll-a fluorescence in cotton genotype Z0102 under different treatments. (**A**) spider plot of fluorescence parameters of cotton leaves, and for each parameter, the value of control was set 1. (**B**) Fv/Fm. (**C**) fluorescence intensity of K point. (**D**) PIabs, performance index for energy conservation from photons absorbed by PSII until the reduction of intersystem electron acceptors. (**E**) Psi (Eo), the ratio of QA driven by exciton occupancy in excitons captured by the reaction center to reduce excitons to drive electron transfer to other electron acceptors in the electron transport chain beyond QA. Different letters mean significantly different among treatments at *p* ≤ 0.05.

To quantify and analyze the changes of the main components related to the structure and function of the photosynthetic electron transport chain under various treatments, the JIP test was used to calculate and derive the relevant parameters from the OJIP curve. As represented in the spider plot, most of the values of the parameters characterizing PSII functioning showed a wide difference in salt-treated cotton seedlings regardless of the exogenous Si, compared to the seedlings under normal conditions (Figure 9A and Figure 10A).

The maximum quantum yield Fv/Fm of PSII primary photochemistry can be used to assess the severity of plant biotic and abiotic stresses. Fv/Fm of non-Si pretreated seedlings under NaCl stress reduced by 36.6% in Z0102 and 22.4% in Z9807, compared with their control plants. The application of Si alleviated the adverse effect of NaCl as the Fv/Fm value increased by 17.3% to 41.1% as compared to the NaCl treatment (Figure 9B and Figure 10B). Similarly, the performance index on the absorption basis (PIabs) was also reduced under salinity stress and can be improved by Si (Figure 9C and Figure 10C).

The appearance of the *K* phase indicates that the donor side of PSII is damaged, which is mainly related to the oxygen evolving complex (OEC). The fluorescence intensity of the K-point of salt-treated plants notably increased in both genotypes, and it was decreased by the application of Si (Figure 9A,C and Figure 10A,C), indicating that Si could alleviate the damage of salt stress to the OEC. Psi (Eo) is the ratio of the QA driven by exciton occupancy in excitons captured by the reaction center to reduce excitons to drive the electron transfer to other electron acceptors in the electron transport chain beyond the QA, enabling it to reflect the light energy transfer efficiency of the PSII reaction center intuitively. It is obvious that the psi (Eo) of these two varieties decreased significantly under NaCl treatment, but the application of Si could improve this index effectively (Figure 9E and Figure 10E).

### 3.10. Si Enhanced the Activities of Key Enzymes in Photosynthesis and the Expression Levels of RubsiCO Genes in Cotton Seedlings under Salt Stress

**Figure 11:**
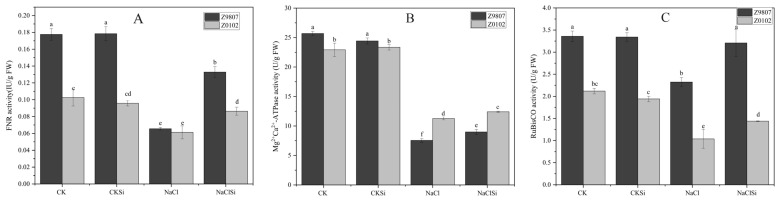
Effects of Si on the activity of enzymes related to photosynthesis. (**A**) FNR activity; (**B**) Mg^2+^Ca^2+^-ATPase activity; (**C**) RubisCO activity. Bars represent the mean ± SD (*n* = 3). FNR, ferredoxin-NADP oxidoreductase; RubisCO, ribulose-1, 5-bisphosphate carboxylase/oxygenase. Different letters mean significantly different among treatments at *p* ≤ 0.05.

In order to further explore the reason why silicon increases the photosynthetic rate of cotton under salt stress, we compared the changes of key enzyme activity in the photosynthesis under different treatments (Figure 11). Ferredoxin-NADP oxidoreductase (FNR) is responsible for catalyzing the generation of NADPH (nicotinamide adenine dinucleotide phosphate) in photosynthetic linear electron transfer, providing a reducing agent for the carbon reaction of photosynthetic. As shown in Figure 11A, the FNR activity of cotton leaves treated with NaCl was significantly lower than other treatments. Exogenous Si significantly enhanced FNR activity under salt stress. Chloroplast ATP synthase catalyzes ATP synthesis, which provides energy for the carbon reaction of photosynthesis. Similar to FNR, salt stress also caused a significant reduction in ATPase activity, and Si prevented the decline (Figure 11B). RubsiCO is the rate-limiting enzyme for photosynthesis. In the presence of NaCl, the activity of RubsiCO decreased remarkably in both cotton genotypes, especially the salt-sensitive Z0102 (Figure 11C). Si pretreatment significantly alleviated the decrease in RubisCO activity caused by salt stress in both cotton genotypes (Figure 11C).

**Figure 12:**
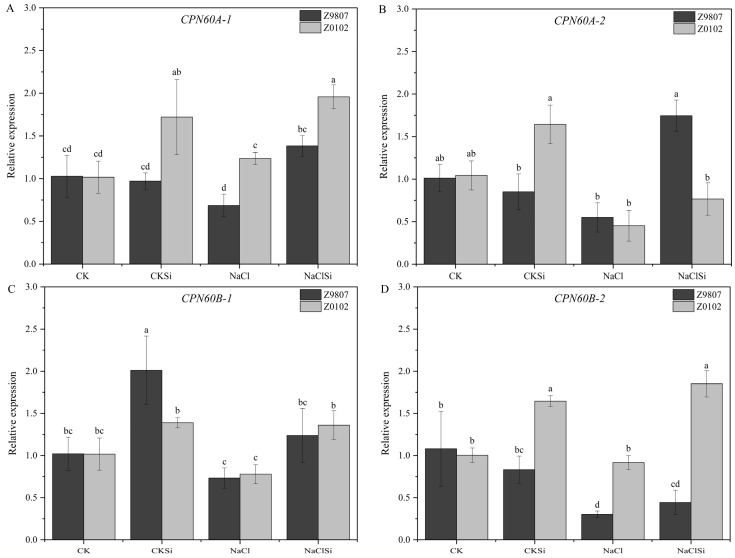
Effects of Si on transcript levels of RubisCO genes. (**A**) relative expression of *CPN60A-1*; (**B**) relative expression of *CPN60A-2*; (**C**) relative expression of *CPN60B-1*; (**D**) relative expression of *CPN60B-2*. Vertical bars represent ±S.E. (*n* = 3). Same letter means not different among treatments at *p* ≤ 0.05. Different letters mean significantly different among treatments at *p* ≤ 0.05.

Expression levels of genes involved in the biosynthesis of RubisCO, *CPN60A-1*, *CPN60A-2*, *CPN60B-1*, and *CPN60B-2* were evaluated at the transcriptional level using qRT-PCR (Figure 12). Overall, salt stress did not significantly affect the expression of RubisCO genes, except that the expression level of *CPN60B-2* in the Z9807 cultivar was significantly down-regulated. Under salt stress, Si pretreatment significantly up-regulated the expression of *CPN60A-1* in both cotton genotypes, but for *CPN60B-1* and *CPN60B-2*, exogenous Si only significantly up-regulated its expression in Z0102. Under salt stress, the expression level of *CPN60A-2* in Z9807 seedlings treated with Si was 3.2 times higher than that in non-Si treatment, but there was no significant difference between the two treatments in Z0102.

## 4. Discussion

Although a large number of studies have shown that Si plays an effective role in improving crop resistance to stress, most of them focus on Si accumulators like wheat, barely, rice, and maize, whereas fewer reports were on crops with low Si accumulation [22]. Here, our results showed that cotton seedlings pretreated with Si could grow better under salinity conditions compared with no Si pretreatments (Figure 1). The present study proved that Si can improve cotton tolerance to salt stress, even though cotton belongs to low-Si accumulation crops [12].

### 4.1. Si Alleviates Salt Damage by Modulating the Improvement of Photosynthesis

Photosynthesis provides material and energy for plant growth and is very sensitive to environmental stress. Salt stress usually causes a significant decrease in the rate of photosynthesis and leads to plant growth inhibition [19]. Si has been proven to be able to mitigate the adverse effect of environmental stress on photosynthesis in many plant species [20,35]. The possible mechanisms for Si-induced improvement of photosynthetic efficacy are mainly involved in the following aspects: (1) Si could improve stomatal function and alter gas exchange relations, thus enhancing the photosynthesis rate. Exogenous Si can improve the stomatal conductance under saline conditions in alfalfa [36]. Similar results were also found in okra and cherry tomato [26,37]. Si supplementation induced an increase in the stomata density and stomata size, thereby contributing to the Gs improvement [37]. In addition, the application of Si could increase the expression of proteins that control the movement of stomatal aperture [38], increase the leaf water level, and keep the stomata open under stress conditions [39]. (2) Si mitigates the degradation of photosynthetic pigments, damage of thylakoid structure, and the decline in photosynthesis-related enzyme activities, etc., and the adverse effects caused by abiotic stress including salinity [10]. Li et al. [20] reported that salt stress caused an obvious decrease in chlorophyll content, whereas the decline was prevented by the addition of Si in the tomato. Cao et al. [40] showed that Si effectively reduced the damage of the chloroplast ultrastructure of the tomato leaves under drought stress, and the protective effect of Si on chloroplasts was also confirmed in other crops [10,41]. In the current study, Si-induced increases in stomatal conductance and chlorophyll content, improvement of thylakoid integrity, an increase in key enzyme activities (FNR, ATPase and RubisCO), and the up-regulated expression level of RubsiCO genes were observed (Figure 6, Figure 7, Figure 10, Figure 11 and Figure 12, Table 2), indicating the positive effect of Si on cotton photosynthesis under salt stress both through adjusting stomatal factors and non-stomatal factors. Furthermore, our present study showed that the Ci, Gs, and Tr were significantly decreased by salt, and the application of Si significantly improved these parameters (Table 1), indicating that Si-induced improvement in photosynthesis containment was mainly related to stomatal factors [42]. Meanwhile, the increase in effective stomata density and stomata opening also confirmed this (Table S2). Nonetheless, the mechanism on how Si regulates stomata response remains unclear and needs more investigation.

### 4.2. Si Alleviates Salt Induced Oxidative Stress by Promoting the Effective Transport of Photosynthetic Electrons and Regulating the Activities of Antioxidant Enzymes

It is well noted that chlorophyll fluorescence is closely related to preliminary photosynthetic reactions. When the plant is subjected to stress, the change of chlorophyll fluorescence parameter is an effective index that reflects the primary reaction alternations of PSII [43]. PSII is located on the chloroplast thylakoid membrane and is an important part of the photosynthetic electron transport chain; the main binding sites of photosynthesis and the orderly transmission of the photosynthetic electron chains are all controlled by PSII [44]. In this study, salt stress significantly reduced the Fv/Fm, whereas the reduction was notably eliminated by the pretreatment with Si (Figure 9B and Figure 10B). Similarly, the PIabs was also improved by Si application with the presence of NaCl (Figure 9C and Figure 10C), indicating a positive role of Si in promoting the primary photochemical reactions of PSII in the waterside. These results were consistent with previous studies reporting that Si could mitigate the adverse effects of salt on photosynthesis and plant growth through enhancing the photochemical efficiency of PSII [36,45]. The oxygen-evolving complex on the PSII donor side is an important part of the PSII core complex and contributes to the catalytic decomposition of H_2_O, which converts the light energy into chemical energy. The appearance of K-point in the fluorescence curve indicates that the oxygen-evolving complex is significantly damaged [46]. Furthermore, the degree of destruction of the oxygen-evolving complex can be calculated by comparing the magnitude of the K-point fluorescence intensity by the JIP-measurement data processing method [47]. In this study, salt stress significantly increased the K-point fluorescence value, which was consistent with the results of Xu et al. [45] in maize. The application of the Si fertilizer markedly lowered the K-point fluorescence value of the salt-stressed cotton seedling (Figure 9D and Figure 10E), indicating that Si can alleviate the damage of oxygen-evolving complexes caused by salt stress to a certain extent, and relieve the suppression of the photosynthetic electron production and transport due to salt stress [44,45]. Further, the Psi (Eo) was significantly lower in the salt-treated seedling (Figure 9E and Figure 10E). This demonstrated that salinity could decrease the electron transfer efficiency of PSII, thus causing a decline in PSII active reaction centers [48,49]. Under the saline condition, the cotton seedling treated with Si showed a lower Psi (Eo) compared with the no Si-pretreated seedlings (Figure 9E and Figure 10E). Taken together, the addition of Si promoted the light energy utilization as well as the electron transfer efficiency, and improved the stability of PSII and, therefore, protected the PSII from salt-induced damage. Consistent with this finding, the improvement of functional units of photosynthetic electron transport chains in oat under salt stress have been reported to be related to exogenous calcium regulation [50].

It is well known that salt stress usually induces oxidative damage in plants. The over accumulation of reactive oxygen species (ROS) can cause fatal oxidative damage to the protein, deoxyribonucleic acids, lipids, and other important biomolecules [23], thereby restraining the growth and development of plants. Chloroplast is one of the major sources of ROS such as single oxygen (^1^O_2_), O_2_^−^, and H_2_O_2_. When plants are exposed to light, excessive energy in the antenna system or reaction center in PSII will induce the production of a large amount of highly destructive ^1^O_2_. In addition, high salt, drought, and other stresses cause stomata closure, resulting in insufficient CO_2_ available and the generation of ^1^O_2_ [51]. ETC on PSI and PSII are the main sources of ROS in chloroplasts [52] Normally, electrons flow from the active PS center to NADP (nicotinamide adenine dinucleotide phosphate), reducing it to NADPH, which then enters the Calvin cycle and reduces the final electron acceptor, CO_2_. However, when NADP is in short supply due to stress, ETC overloads and electron leakage occurs, and thus ROS is produced [51]. Our present results showed that the accumulation of H_2_O_2_ and O_2_^−^, as well as MDA content, were significantly higher in salt-treated cotton leaves compared with the control (Figure 3 and Figure 4), which indicates oxidative damage caused by salt. Exogenous usage of Si remarkably lowered the amounts of ROS and MDA by improving the antioxidant enzyme and photosynthetic electron transport of the salt-treated cotton (Figure 4, Figure 9 and Figure 10). Consistent with our study, Saleh et al. [53] reported that the application of Si considerably enhanced the activity of SOD and CAT in salt-treated wheat, followed by an induced expression of genes related to these enzymes [54]. Thorne et al. [22] also reported that Si could reduce oxidative damage by an average of about 30% under both drought and salinity stress. In the present study, Si application significantly reduced the accumulation of ROS and its damage to substances, enzymes, and structures related to photosynthesis in salt-stressed cotton, thereby maintaining a relative normal plant growth under salt stress. According to these findings, a protective mechanism for Si in the alleviation of salt stress is by mitigating the salt-induced oxidative damage.

## 5. Conclusions

Altogether, this study involved integrated physiology, electron microscope observation, and gene expression analyses in exploring the mechanisms of Si-induced tolerance to salt stress in cotton. In the present study, salt stress caused an increase in ROS levels, mainly due to decreased antioxidant enzyme activity and photosynthetic capacity. Si could mitigate the adverse effects of salt stress mainly by (i) up-regulating the expression of antioxidant enzyme genes and enhancing the activity of antioxidant enzymes, and (ii) improving the photosynthetic rate and electron transfer efficiency via protecting the thylakoid membrane structure, enhancing the activity of PSII, increasing the activity of key photosynthetic enzymes, and maintaining the opening of the stomata. These findings might help to better understand the theoretical basis for adding exogenous silicon to improve the plant tolerance to salt.

## Figures and Tables

**Table 1:** Effect of exogenous Si on gas-exchange parameters of cotton leaf under saline and non-saline conditions.

Genotype	Treatment	Pn (µmol m^−2^ s^−1^)	Ci (µmol mol^−1^)	Gs (mol m^−2^ s^−1^)	Tr (mmol m^−2^ s^−1^)
Z9807	CK	8.2 ^a^	322.7 ^b^	0.20 ^b^	3.35 ^a^
	CKSi	8.1 ^a^	332.9 ^b^	0.23 ^b^	3.80 ^a^
	NaCl	5.3 ^c^	223.3 ^d^	0.05 ^e^	1.20 ^d^
	NaClSi	7.3 ^b^	266.8 ^c^	0.09 ^c^	1.60 ^c^
Z0102	CK	7.4 ^b^	329.1 ^b^	0.20 ^b^	2.70 ^b^
	CKSi	7.2 ^b^	347.3 ^a^	0.28 ^a^	3.61 ^a^
	NaCl	4.7 ^d^	220.3 ^d^	0.06 ^e^	1.12 ^d^
	NaClSi	7.0 ^b^	278.3 ^c^	0.11 ^c^	1.96 ^c^

Pn, net photosynthesis rate; Ci, intercellular CO_2_ concentration; Tr, transpiration rate; Gs, stomatal conductance. Data represents means of three replicates. Different letters within same testing trait indicates significant differences between treatments (*p* ≤ 0.05).

**Table 2:** Effects of Si on photosynthetic pigments of cotton leaf under saline and non-saline conditions.

Genotype	Treatment	Chlorophyll Content (mg g^−1^ FW)			Carotenoids (mg g^−1^ FW)
		Chlorophyll a	Chlorophyll b	Chl (a + b)	
Z9807	CK	1.54 ^a^	0.84 ^ab^	2.37 ^a^	0.083 ^b^
	CKSi	1.53 ^a^	0.88 ^a^	2.41 ^a^	0.102 ^a^
	NaCl	0.92 ^d^	0.52 ^de^	1.44 ^d^	0.063 ^cd^
	NaClSi	1.29 ^b^	0.66 ^c^	1.95 ^b^	0.066 ^cd^
Z0102	CK	1.27 ^b^	0.78 ^b^	2.05 ^b^	0.075 ^bc^
	CKSi	1.27 ^b^	0.78 ^b^	2.04 ^b^	0.087 ^b^
	NaCl	0.75 ^e^	0.49 ^e^	1.23 ^e^	0.054 ^d^
	NaClSi	1.05 ^c^	0.56 ^d^	1.61 ^c^	0.065 ^cd^

Data represents means of three replicates. Different letters within same testing trait indicates significant differences between treatments (*p* ≤ 0.05).

## Data Availability

The data is contained within this article and Supplementary Files.

## Supplementary Materials

The following supporting information can be downloaded at: https://www.mdpi.com/article/10.3390/antiox11081520/s1, Table S1: Primer sequences used for qRT-PCR; Table S2: Effect of exogenous silicon on stomatal characteristics of Z9807 and Z0102 under saline and non-saline conditions.
