# Supplementary material for: Ameliorative Effects of Silicon against Salt Stress in Gossypium hirsutum L
Source: Antioxidants (Basel). 2022 Aug 4;11(8):1520. doi: 10.3390/antiox11081520 (PMC9404900; doi:10.3390/antiox11081520)
Supplement: Supplementary file 1 [file antioxidants-11-01520-s001.zip › High lights.pdf]

1. Si can mitigate the negative effect of salinity on cotton growth.
2. Si decreased the ROS level of salt stressed cotton plants.
3. Si enhanced the antioxidant enzyme activity of salt-stressed cotton.
4. Si alleviated the decrease of electron transfer rate caused by salt stress.
