# Supplementary material for: Ameliorative Effects of Silicon against Salt Stress in Gossypium hirsutum L
Source: Antioxidants (Basel). 2022 Aug 4;11(8):1520. doi: 10.3390/antiox11081520 (PMC9404900; doi:10.3390/antiox11081520)
Supplement: Supplementary file 1 [file antioxidants-11-01520-s001.zip › Table S.pdf]

Table S1 Primer sequences used for qRT-PCR.

gene ID	Genen Name	Forward primer (5′ -3′)	Reverse primer (5′ -3′)
Gh_A13G2023	CNP 60A-1	CGAGGGTGCTAGTAACTGAT	TCCAGTAACATCCTCCGCAA
Gh_D13G2425	CNP 60A-2	CGAAAGTAGTCAATGATGGG	CCATCACCTGCAGAGTCATT
Gh_A05G1083	CNP 60B-1	TGGCTATAATGCTGCAACTGG	GATGTCGACAACCACGGCAT
Gh_D05G1261	CNP 60B-2	CTAGTAGATGTGGCTGCTGT	TTGTTTTCCGAACTGCTACCT
Gh_A01G0845	CAT1	TGCATTTTGCCCTGCCATTGTG	TGTGCCTCTGGGTATCAGCGTA
Gh_D06G1170	POD	TCAAGAGGCTGTCAATCTGGCG	TGGCCAGTGTTCGATTTGCTGT
Gh_D13G1062	SODCC	ACTCAAGAGGGAGATGGCCCAA	AATGGAGTGTGGCCCAGAGAGA
Actin	—	ATCCTCCGTCTTGACCTTG	TGTCCGTCAGGCAACTCAT

Table S2 Effect of exogenous silicon on stomatal characteristics of Z9807 and Z0102 under saline and non-saline conditions

Genotype	Treatment	Length (μm)	Width (μm)	Surface (μm^2)	ESD (No. mm^{-2})
Z9807	CK	14.33 ^{c}	6.41 ^{a}	14.65 ^{b}	397.7 ^{a}
	CKSi	15.04 ^{b}	6.41 ^{a}	15.70 ^{b}	386.4 ^{a}
	NaCl	13.07 ^{d}	6.51 ^{a}	4.06 ^{e}	181.8 ^{d}
	NaClSi	14.94 ^{b}	6.35 ^{a}	12.14 ^{c}	340.9 ^{b}
Z0102	CK	14.25 ^{c}	6.31 ^{a}	15.02 ^{b}	431.8 ^{a}
	CKSi	15.99 ^{a}	6.38 ^{a}	17.18 ^{a}	375.0 ^{a}
	NaCl	13.11 ^{d}	6.51 ^{a}	2.99 ^{e}	159.1 ^{d}
	NaClSi	15.22 ^{b}	6.31 ^{a}	7.01 ^{d}	250.0 ^{c}

ESD, Effective stomata density (number of opening stomata mm^{-2}). Data represents means of three replicates. Different letters within same testing trait indicates significant differences between treatments ($p \leq 0.05$).
